# An exploration of opportunities and challenges facing cervical cancer managers in Kenya

**DOI:** 10.1186/1756-0500-6-136

**Published:** 2013-04-08

**Authors:** Lucy W Kivuti-Bitok, Ganesh P Pokhariyal, Roudsari Abdul, Geoff McDonnell

**Affiliations:** 1 School of Nursing Sciences, University of Nairobi, P.O BOX 19676-KNH-00202, Nairobi, Kenya; 2 Health and Information Science, University of Victoria, PO Box 3050 STN CSC, Victoria, BC, V8W 3P5, Canada; 3 School of Mathematics, University of Nairobi, P.O Box 30196-GPO-00100, Nairobi, Kenya; 4 Centre for Health Informatics, University of New South Wales, Sydney, NSW, 2052, Australia

**Keywords:** Challenges, Attitudes, Opportunities, Cervical cancer, Health care managers, Kenya

## Abstract

**Background:**

Kenya like other developing countries is low in resource setting and is facing a number of challenges in the management of cervical cancer. This study documents opportunities and challenges encountered in managing cervical cancer from the health care workers’ perspectives. A qualitative study was conducted among cervical cancer managers who were defined as nurses and doctors involved in operational level management of cervical cancer. The respondents were drawn from four provincial hospitals and the only two main National public referral hospitals in Kenya. Twenty one [21] nurse managers and twelve [12] medical doctors were interviewed using a standardized interview guide. The responses were audio recorded, transcribed verbatim and the content analyzed in emerging themes.

**Findings:**

Four themes were identified. Patient related challenges included a large number of patients, presenting in the late stage of disease, low levels of knowledge on cancer of the cervix, low levels of screening and a poor attitude towards screening procedure. Individual health care providers identified a lack of specialised training, difficulty in disclosure of diagnosis to patients, a poor attitude towards cervical cancer screening procedure and a poor attitude towards cervical cancer patients. Health facilities were lacking in infrastructure and medical supplies. Some managers felt ill-equipped in technological skills while the majority lacked access to the internet. Mobile phones were identified as having great potential for improving the management of cervical cancer in Kenya.

**Conclusion:**

Kenya faces a myriad of challenges in the management of cervical cancer. The peculiar negative attitude towards screening procedure and the negative attitude of some managers towards cervical cancer patients need urgent attention. The potential use of mobile phones in cervical cancer management should be explored.

## Background

Cervical cancer in Kenya is the second most frequent cancer after breast cancer and accounts for an average of 2000 deaths per year [1]. Each year a crude incidence rate of 16.5 per 100,000 women and age standardized rate of 28.7 is reported [1]. It is important that Kenya as a country puts preventive and intervention measures in place in order to deal with the disease burden from cervical cancer. Kenya, like other developing countries, is low in resource setting and is facing a number of challenges in managing cervical cancer.

Makin et al. [2] identified challenges associated with screening, ranging from low levels of cervical cancer screening due to poor access to organized screening, a lack of or low information on cervical cancer screening, women’s perception of low threat of disease and over-burdened health care facilities which lack equipment and are understaffed. Bingham et al. [3] found that ineffective infrastructure as well as long distance between facilities and clients’ homes increase transportation costs and delay reporting results. Cervical cancer screening is associated with high costs which may not be affordable to most women [4]. At the Kenyatta National Referral Hospital, pap smear costs Kshs 550(Approx $7) . However, the client must pay for a file or identification card of Kshs 500(Approx$ 6.5). The client then needs to wait for the pap results from the pathologist, which takes two weeks. At the leading private hospitals, Pap smear costs on average Kshs 1200($15) with a waiting time of 2 days for the pathology results. However at the public Hospitals, the Pap smear procedure is subsidized by the government and the client only pays Kshs 20 (Approx $0.25) for registration. The waiting period is however two weeks. Public Hospitals also make use of Visual Inspection with Acetic Acid (VIA) and Visual Inspection with Lugol’s Iodine (VILLI) as screening methods in low resource settings and results are available immediately. Of the few women who undergo screening, a large portion of them do not return for their test results [5].

Staffing has been identified as a challenge in the screening and management of cervical cancer. Even though Chirenje et al. [6] reported that there was an adequate number of staff for cervical cancer screening services, these personnel may not have had the required screening skills or equipment.

Chirenje et al. [6] however argue that health care institutions in east and central Africa have the necessary infrastructure for cervical cancer screening, but these facilities experience frequent shortages of materials needed for taking Pap smears.

Human Papilloma Virus [HPV] Vaccination, on the other hand, is expensive and may not be affordable to most women in developing countries. There is limited knowledge and information on HPV vaccination.

Health Information management is a challenge. There is an evident lack of timely cancer registries with incomplete risk factors. This poor quality data curtail reliable population based estimates for incidence rates, mortality rates and the effectiveness of interventions [7]. Other challenges faced as a result of health system deficiencies include: limited training among health care providers, lack of resources and poor data management systems [8].

While a number of studies have focused on challenges faced by the patients in cervical cancer management [2], [6], few have focused on the challenges faced by the health care workers.

It is important to establish the challenges faced by cervical cancer managers and identify ways of dealing with these challenges to improve health care system performance.

This study aimed at exploring opportunities and challenges identified by cancer managers both at peripheral (provincial) and tertiary (National/ referral hospitals) with the view of gaining a deeper understanding of the system. The study focused on Information communication and Technology (ICT), service delivery and screening issues.

## Findings

Respondents were drawn from the only two National referral hospitals; Kenyatta National Hospital [KNH] with a bed capacity of 2500 and Moi Teaching and Referral Hospital [MTRH] with a bed capacity of 800. KNH houses the sole radiotherapy facility in the public sector and receives patients with different types of cancer from all over the country. The two hospitals receive over 70% of cervical cancer patients from all over the country.

Fifty percent (50%) of the Eight Provincial hospitals were randomly sampled. North Eastern Province was however conveniently excluded from the study, due to insecurity being experienced at the time. The provincial hospitals were: Kisumu Provincial General Hospital [PGH] located in the western region/Nyanza province with a bed capacity of 457, Coast PGH with a 499 bed capacity, Nakuru PGH located in the Rift Valley Province which has a bed capacity of 588 while Embu PGH has a bed capacity of 618 and is located in the Eastern Province of Kenya. Each of the provincial hospitals has a Mother Child Health and Family Planning Clinic (MCH/FP), a medical or Gynecology ward which admits cervical cancer patients. Each of the wards and clinics has an average of 5–20 nurses working in the Unit while each is headed by one to two Nurse Managers and each Provincial Hospital has 1–3 Medical Officers serving the Units. Each of the referral Hospitals has approximately 100 Nurses and 10 to 20 Medical Doctors providing care to cervical cancer clients.

### Design

This Qualitative study was carried out for a period of four months (April 2012 to July 2012). A purposive sample of all operational level Nurse Managers (NM) who were included in charges of gynecological and oncology wards admitting cervical cancer clients and radiotherapy departments were interviewed. The Medical Doctors (MD) who were managing patients in these wards and at radiotherapy departments were also interviewed. The researchers believed that this group of cervical cancer managers were the operational level managers and were also involved in the day to day management of the cervical cancer clients and hence were best suited to provide a balanced account of their experiences.

Ethical clearance was obtained from the Kenyatta National Hospital/University of Nairobi [KNH/UON] ethics and research committee, the National Council of Science and Technology [NCST] as well as individual hospitals where the study was conducted. The participants had full disclosure of information. Written consent was obtained from all participants, respondents did not identify themselves by names during the interviews to ensure anonymity and confidentiality. A pre-test was done at the Hospice of Kenyatta National Hospital, among three nurse managers and two Medical Doctors with a Cronbach’s alpha of 0.71. An interview guide (Additional file 1: Appendix 1) was used to ensure all coverage of key issues identified, which also included use of internet and mobile phones among other things.

Interviews were conducted by the principal investigator and one research assistant who was a holder of an MSc in Nursing. They were trained in qualitative research methods and specifically in conducting interviews with health care workers.

The order of the discussion was guided by the interview guide. The respondents were to raise other issues they deemed important in the management of cervical cancer. Individual interviews lasted 30 to 60 minutes and took place at the doctors’ or/and nurses’ office within the work areas. Interviews were audio recorded. The audio recording of the interviews allowed eye contact between the interviewers and the respondents, as well as obtaining accuracy from the recordings rather than relying on memory or field notes.

To increase the validity of the interviews, the audio recordings were played back to the respondents and they were allowed to make alterations of their statements where need arose. However none of the respondents felt the need to change their recorded statements.

The researchers listened to the audio recordings and compared them with the transcripts in order to confirm and ensure that the two were identical. The recorded interviews were transcribed to identify emergent themes. The verbatim transcription avoided complex statistical analysis. Key themes were described using direct quotes from the respondents against their profession.

A total of thirty three (33) respondents were interviewed comprising 12(36%) medical doctors and 21(64%) nurse managers. This was a response rate of 73%. This response rate did not affect the results of the study, as saturation level was reached and further interviews yielded no new findings. This sample size was deemed sufficient based on the recommended sample size of 12 interviews in qualitative studies by Guest et al. [9]. The respondents were basically homogenous, were professionals and generally possessed a degree of expertise in their field. Reasons given for non-participation were: being away on other assignments; having a large workload; while one nurse manager was uncomfortable with being audio recorded. Thirty three percent (n=11) were from the National referral hospitals and 67% (n=33) from peripheral hospitals. The average age of the respondents was 41.1 years with a standard deviation (SD) of 5.6. The average length of experience of working with cervical cancer was 7.29 year with SD 5.4.

The nurses’, managers’ and doctors’ distribution and biographical characteristics were as indicated in Table 1.

**Table 1:** Biographical characteristics of cervical care managers

**Workstation**	**Sex**			**Profession**			
	**Females**	**Males**	**Total**	**Medical doctors**	**Nurse managers**	**Total**	**Percentage**
Coast Provincial General Hospital	2	2	4	1	3	4	12.1
Embu Provincial General Hospital	4	1	5	2	3	5	15.2
Kenyatta National Hospital	4	4	8	3	5	8	24.2
Kisumu Provincial General Hospital	3	2	5	1	4	5	15.2
Moi Teaching and Referral Hospital	4	3	7	2	5	7	21.2
Nakuru Provincial General Hospital	1	3	4	3	1	4	12.1
**Totals**	18	15	33	12	21	33	100

**Figure 1:**
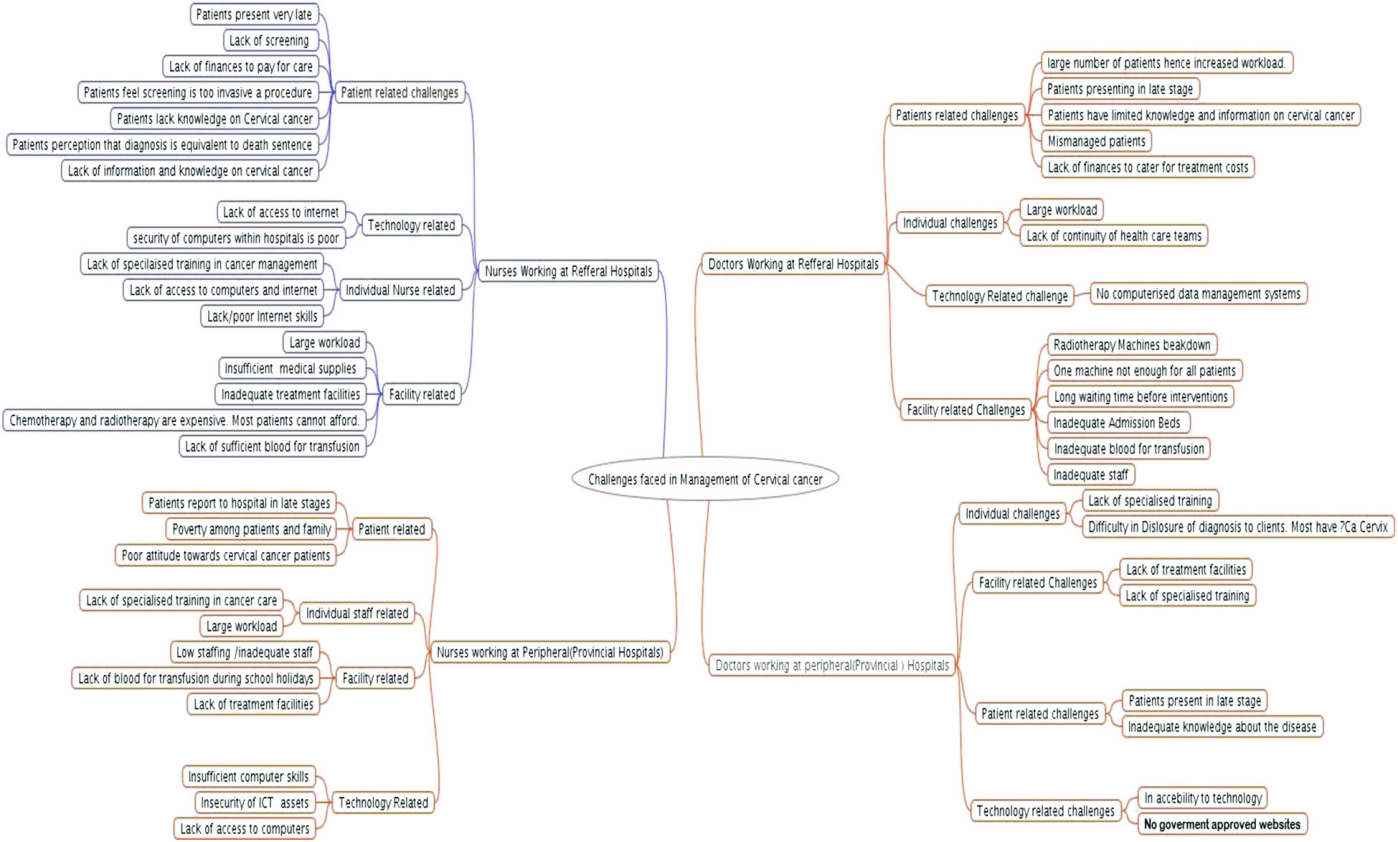
Summary of challenges faced by cervical cancer manager.

The four themes which emerged were patient related challenges, individual health care provider challenges, health facility related challenges and technology related challenges. Challenges identified were similar except that peripheral hospitals did not have challenges in getting blood for transfusion, while KNH faced challenges with breakdown of the radiotherapy machine. Figure 1 is a summary of the key challenges identified.

### Patient related challenges

These were identified and included large workload, presenting in late stage, limited knowledge about cervical cancer, poor attitudes of some health care providers and relatives towards the patients and poor attitudes towards cervical cancer by patients themselves.

### Large workload

The Cervical cancer managers reported taking care of a large number of patients. Large workload hinders quality of care provided to the cervical cancer patient. Both nurses and doctors equally reported feeling overwhelmed with the large number of patients.

‘It is overwhelming. We get 5–10 patients a day’ [NM- 6]

‘*The patients are so many, the gynecologists see the patients but the oncologists do not get to see most of these patients’* [NM-4].

The increase in the number of clients was also attributed to a long waiting time before clinical intervention.

‘These patients are chronic patients, once they land in the ward, we are meant to stabilize them for radiotherapy. Bed occupancy goes higher because stabilizing the patients takes longer’ [NM-2].

### Inadequate knowledge about the disease

The managers reported receiving patients who had limited knowledge about cervical cancer. Lack of awareness of disease signs and symptoms as well as interventions available were mentioned as a challenge.

‘Many women are not aware of cervical cancer; the government needs to step up health education and campaigns about cancers in general’ [NM-8].

‘Even the nurses themselves have the knowledge and are not using the knowledge. How to integrate knowledge and practice is a problem. Let the survivors speak to the other because they have moral courage’ [NM-11].

Health care managers were of the opinion that education of the population about cervical cancer should not be the responsibility of the government alone. They felt that use of mobile phones could reach a wider coverage in health education even as an initiative of the health facilities and health care workers.

‘Almost every homestead has a mobile phone nowadays. Even if the women do not know how to read sms, we could use voice messages in their local language to educate them’ [NM 12].

There was a feeling that knowledge of cervical cancer screening procedures may have a negative effect on the clients seeking screening services

‘Too much knowledge may also have a negative effect on screening. The scraping of cervical cells creates a wound which my act as entry point for other infections including HIV. Once many women realize this, they may decline to have the screening procedure done’ [NM-19].

### Low screening levels and poor attitude towards cervical cancer screening procedure

Low screening coverage is a big challenge for the health care workers. Even though screening is integrated in the Family Planning clinics and Maternal Child health care units, many felt that more can be done to mobilize women to seek screening services. Use of mobile phones was identified as a big opportunity in campaigning for screening.

‘Whoever is in health facilities has contacts of the phones. If even us we used contacts of 50% of women you have on phone and tell them to come for screening the impact would be great’ [NM-16].

Health care workers reported that many clients find the screening procedure too invasive and is viewed as embarrassing and against the African culture. Some women find it culturally unacceptable to have young nurses and doctors see their private parts. Others felt that it was culturally unacceptable to allow a male to see their private parts. Even among health care workers, screening is still seen as an uncomfortable procedure associated with risk of infections.

‘Many women feel embarrassed about the screening procedure. Even my fellow health care workers find it too invasive’ [MD-4].

*‘It’s difficult because, not many will say that I have a problem in my genital area. They will not encourage a man especially to view their private parts’ [NM-19]*.

‘Others have a problem with exposing their bodies to the young doctors and nurses, who they view as their children’ [NM- 23].

‘Even I with all my medical Knowledge find it hard to expose my body every three years. I am also suspicious of HIV infection from the use of speculum. I am not sure of the sterility methods they use’ [NM-18].

‘Disposable speculums are expensive and their supply may not be sustainable in Government facilities’ [NM-18].

One of the health managers suggested vaccination as an alternative measure to screening.

‘Screening in itself has been found to be effective in developed countries. However, our people do not like the procedure and their health seeking behavior is dictated a lot by their cultural beliefs. Even though vaccination is expensive, it may be more beneficial for us as a country’ [MD-6].

### Patients presenting in late stage

Many of the patients present in a late stage of cervical cancer. This was attributed to a lack of proper diagnosis at the peripheral facilities as well as mismanagement of patients before referral to provincial and tertiary facilities.

‘I have only seen one patient who came with stage one. All the others come after stage 2’ [NM-4].

*‘Majority of patients come at stage three and four’* [*M D- 4].*

‘Being referred as too late a case. People come thinking that they coming to get healing or get cured. It’s very challenging during counseling to tell patients that even though you have come it is too late and there is nothing much we can do’ [MD-6].

### Lack of finances to cater for treatment costs

Inability of the patients to pay for cervical cancer treatment was attributed to the poverty of most patients as well as the high cost of chemotherapy and radiotherapy. Patients lacked health care insurance and only a few were reported to be members of National Hospital Insurance Fund [NHIF]. Since many patients could not afford care, hospital facilities ended up waiving their medical bills hence a loss of revenue to the hospitals.

‘Some come from far and are very poor. I advice them to join NHIF. The bills become a problem. They stay until the hospital waives them’ [NM-6].

‘Without money you cannot do all the things required in time. Even if the doctor would want to treat immediately, Staging changes as patients await money. Patients have to cough money’ [NM −17].

‘Patients can’t afford care. Cancer treatment is expensive. Unless there is a donor, poverty is a major challenge. Those unable to afford just stay home and die’ [MD-10].

‘Those with NHIF card benefit but some can’t even afford the 300 shillings to registers for NHIF’ [MD-13].

‘They may miss treatment as chemotherapy is very expensive. Others may die before they receive treatment. Some have no dependants who are capable’ [NM-6].

### Poor attitude towards cervical cancer patients by the relatives, public and some healthcare workers

Some healthcare workers view cervical cancer patients as a burden. Some are allocated care of the patients as a ‘disciplinary’ measure. Some health care managers utter negative comments about the cervical cancer patients. As one nurse manager reported her experience in accompanying cervical cancer patients to one of the National referral hospitals: t*he patients would cry, smell, stink and nobody would move near them. I would be sent to escort the patients to KNH for radiotherapy. I would find myself travelling to the KNH with public means. The people would move away from us in the Nissan because of the stench! Minute you get to radiotherapy unit and get to consultation room a doctor would ask ‘who is that stinking like the X hospital patients’ [NM 9].*

Other health care managers appreciate the uniqueness of cervical cancer care and view it almost as a calling:

*‘Not many health care professionals are interested in management of cervical cancer or preventive measures. If interested even those at periphery facilities can do something. However much you try to prevent not many people has shown interest in preventing cancer. If they were interested we would be receiving earlier stages. Pap smear can be collected in a dispensary or elsewhere. Not many people have come up to fight against cancer*’ [NM-17].

*‘Cancer management is not just work. You must have passion for the patient’* [MD-12].

Some patients and relatives perceive diagnosis with cervical cancer as equivalent to a death sentence. Some relatives on the other hand abandon the patients in the hospital to die. As reported by one NM-6:

‘We witness abandonment of patients by relatives. I had one client who stayed here for five months. Nobody ever visited her. The day she died, the husband was here at 8.30 am’ [NM-6].

‘So they just bring them here and no active management is being done. They came when they are at the last point of death. Some leave patients in the ward to die ‘[NM-14].

### Individual cervical cancer manager related challenges

Lack of specialized training in cancer care and its management was reported as a challenge faced by health care managers themselves. Many feel ill equipped to manage the condition. A number however reported having been trained in palliative care. This lack of specialised training has been attributed to mismanagement of patients at peripheral levels.

‘Many health personnel are not aware of what cancer is. It takes 6 months to 12 months for referral to take place’ [MD-4].

Other health care managers have had on job training in cervical cancer management. As reported by one doctor

‘*I remember seeing cervical cancer patients when I was still at Medical school. Now I am working as an MO and I am expected to manage cervical cancer in the ward. I have learnt a lot from the more experienced nurses. I think we need an induction course in managing cervical cancer’ [MD-9].*

‘Poor training and development in area of cancer management compromises patient care’ [NM-17].

‘Not many health care providers are trained especially in narcotic analgesics’ [NM-15].

However some nurse managers in referral hospitals reported having received adequate training in cervical cancer care.

‘Nurses are aware of screening and procedures. We had training from Americans. Nurses and doctors can now do screening and treatment procedures e.g. colonoscopy and leep. Pap smear, a primary screening can be done by everybody’ [NM −3].

### Lack of continuity of health care teams

This was attributed to the inadequate numbers of health care workers and frequent change of team members.

‘Every week the teams of doctors change and change the management decisions too’ [NM-6].

### Difficulty in Disclosure of diagnosis to clients

It was reported that it takes a long time before a cervical cancer diagnosis can be confirmed. Many of the patients in the peripheral facilities are ‘suspected’ to have cervical cancer as the medical team awaits confirmatory test results.

*‘Most patients in the ward are query ca cervix. Many do not know their disease conditions’ ***[**MD-4].

### Facility related challenges

Facility related challenges were identified as inadequate treatment facilities, long time of wait before interventions, inadequate inpatient facilities, and lack of blood for transfusion and inadequate number of staff. Inadequate treatment facilities in relation to numbers, location and availability were identified as a major challenge. It was noted that radiotherapy facilities were only available at Kenyatta National Hospital. This facility serves the whole country as well as some patients from the neighboring countries. This one machine is not enough for all the patients. The machine itself is characterized by frequent breakdowns.

‘….mode of management is radiotherapy which is only available at KNH’ [MD-4].

‘The machine is overbooked or unavailable machines. Patients are booked 3–4 months down the line’ [MD-7].

*‘Most come from rural areas where they have no treatment. By the time they come very little can be done’ [*NM-3].

‘*Even though we should not wait and treat immediately we are forced to schedule patients. Scheduling a patient means you are not doing much for them’* [MD-8].

*‘ They either go to KNH or Uganda for radiotherapy’* [NM 18].

Laboratory and theatres facilities were also identified as inadequate.

*‘They come late, staging in theatre takes time, results and treatment can delay*. *Process of biopsy takes a long time’ [MD 9].*

Other facilities have inadequate beds for admission

‘Cancer does not have a real ward. Space is limited’ [NM-6].

*‘These patients are chronic patients, once they land in the ward, we are meant to stabilize them for radiotherapy. Bed occupancy goes higher because stabilizing the patients takes longer*.’[NM-1].

### Insufficient blood for transfusion

It was noted that blood for transfusion was a big challenge in the referral hospitals.

‘Patients come with very low Hb of 1 to 2. No blood available. Within the hospital the blood is not available’ [NM-6].

‘Kenyatta doesn’t not have blood. It may take up to 3 months to raise the hb to 10. Let the peripheral institutions first bring the hb up’ [NM-2].

‘Anemia, getting blood is a challenge and this also causes delay in intervention’ [NM 3].

However lack of blood was a challenge in provincial hospitals only when schools and learning institutions were closed for holidays.

‘Blood for transfusion is a problem when school is closed’ [NM −23].

### Technology related challenges

Cervical care managers expressed their inadequacies in Utilization of ICT in management of cervical cancer. The challenges identified included lack of computerized data management systems, Lack/poor Internet skills, inadequate access to computers and internet, Poor security of computers and other ICT assets within hospitals, inaccessibility to technology as well as lack of government approved websites.

‘Both patients and health care provider need to be computer literate. Only few of us are competent in computer skills’ [NM-19].

‘We need to develop a website that will serve the sub-Saharan Africa’ [NM-9].

‘We cannot have computers yet because there are no burglar proof doors. The computers may be stolen if kept within the wards’ [NM-7].

The managers reported use of internet in continuing education, updating themselves on management protocols and students education.

‘I search on Cases within my wards. I update myself on computers so as to update my students. I Just Google’ [NM-14].

The respondents acknowledged the accessibility of mobile phones by almost all patients who visited the health facilities. They felt that mobile phones could be used to reach a wider population.

*‘even if the woman does not have a mobile phone, at least one person in the family is likely to have a cell phone’* [NM- 17].

Potential use of cell phones was identified in health education, reminder alert; follow up of patients as well as database management.

‘We can use the mobile phones in helping patients set reminders for pain control. An alarm to remind patients to take medication. If not in pain they forget’ [NM-9].

‘We have not used computers yet. Computer system is not well developed. No access to computers at the hospital. Access is through commercial cyber which are expensive and sometimes are congested’ [NM-16].

‘However there is no way of authenticating the information from the internet. You either take it or leave it’ [NM 11].

Even though facilities may not have mobile phones, health care providers extend use of their personal cell phones in management of cervical cancer patient

‘We give the patients personal numbers. It’s a very friendly department. We get attached to our patients. There is now an office number phone. Over the weekend some patients prefer specific persons and may request for specific phone number’ [NM −9].

What is already known about the study?

Challenges faced by health care workers include large workload, low levels of screening, low knowledge levels on cervical cancer management and inadequate facilities

What does this study reveal?

Some cervical cancer managers have negative attitude towards cervical cancer screening procedure and care of cervical cancer clients.

Vaccination is seen as a more culturally acceptable form of cervical cancer prevention measure.

Acceptability of screening and vaccination are important indicators of potential success of cervical cancer management.

Lack of Blood for transfusion is a big hindrance to cervical cancer treatment.

Managers feel inadequate in computer and internet skills Mobile phones have great potential in health education, reminder alert and information management in cervical cancer.

## Discussion

Cervical cancer managers in Kenya are faced with myriad of challenges which influence their view and management of cervical cancer. The challenges ranged from individual, to facility related, patient related challenges as well as technological related challenges. The respondents cited patients having inadequate knowledge about cervical cancer, few patients were screened and inadequate number of health care workers [3], [5], [6], [10], [11].

Cervical cancer screening is a procedure which involves opening the vagina using a speculum with the woman lying on lithotomic position and taking a sample of cells from the cervix. The procedure may be too invasive to privacy to some women. This study found that screening procedure has been viewed negatively by both clients and some health care worker. Cultural, personal and procedural factors were associated with the negative attitudes. Generally the Traditionally African culture dictated that women expose their private parts only to their spouses and female midwives. Age and Sex differences between the Cervical Cancer managers and women who may require cervical cancer screening may be a hindrance to the screening practice. The results support a study done in United Kingdom which revealed that women found the whole practice of cervical cancer screening embarrassing, uncomfortable and too intimate especially in exposing such personal parts of their body [3], [12]. This embarrassment, discomfort and the exposure may be the reason behind low number of women undergoing screening. A number of women dislike the procedure hence acceptability of the procedure by the women is a big factor in success or failure of screening programs. The results of this study however contradicted Claeys [13] who found that women had positive attitude towards screening. Huchko et al. [14] revealed positive attitudes towards cervical cancer screening among HIV positive women in Kenya while Audet et al. [15] found that 84% of rural women in Mozambique were willing to undergo cervical cancer screening.

Vaccination in this study was mentioned as an alternative to screening. It was thought to be more culturally acceptable compared to screening. This supported studies done among women by Becker-Dreps et al. [16] in Kenya and Diangi et al. [17] in Botswana who found high level of acceptability of HPV vaccines but low level of knowledge on HPV Vaccine. The positive attitude towards vaccination may be related to its being less invasive to privacy and less embarrassing. The positive attitude towards HPV vaccine is a strength which the stakeholders would base HPV vaccine on in these countries.

Some Cervical cancer managers reported feeling of inadequate training in management of cervical cancer while others expressed a negative attitude towards screening procedure. This finding concurs with Chirenje et al. [6] who found that health care workers lacked the necesarry skills in cervical cancer management. This may mean that such a Cervical Cancer manager, who feels ill equipped and has a negative attitude towards screening procedure is less likely to encourage women to undergo screening.

Technology related challenges included digital divide characterised by inaccessibility to hardware as well as internet. Cervical cancer managers also reported feeling of inadequate computer skills. This has the implications that the Cervical cancer managers may not have access to research results or evidence based practice. Their knowledge levels on cervical cancer may also not be uptodate. Few public hospitals are computerised in Kenya hence the medical staff generally do not have access to a computer at the work place. Investment in ICT in most government facilities in Kenya is still rudementary. At the same time, it was not compulsory to have ICT trainig as a component of both nursing and Medical curriculums in the yester years hence with a mean age of 7.9 years of expericnce in cervical cancer management , these health care providers may not have trained in computer skills. Those who have computer skills trained on their own volution. The findings of this study concurs with Bukachi and Pakenham [18] who found low computer skills, inadequate access and uncordinated ICT investment in developing countires. The managers noted the potential use of mobile phones in management of Cervical cancer. This may be attributed to most of the clients having access to a mobile phone atleast at the family level. The potential impact of use of mobile phone in cervical cancer management supports Gormley et al. [19] who indicated that mobile phones could be useful in remote access to specialist for diagnosis and direction on management of diagnised cervical cancer in Botswana.

### Limitations of the study

Purposive sampling in its self has inherent selection bias hence generalization of the results is limited. Three (3) of the researchers have a Medical background hence may have influenced the formulation of the research questions. To eliminate bias, an independent person who was not part of the research team reviewed the questionnaire.

Even though cervical cancer care is also offered at the public Health Institutions of lower levels; from Dispensary (Level 1) to District Level 4 hospitals, this study focused on the National referral Hospitals and provincial Hospitals. It would have been enriching to involve the lower level health facilities. This was however not possible due to limited resources.

### Strengths of the study

This verbatim transcription avoided complex statistical analysis. The thirty three (33) interviews was sufficient given the aim was to understand and document challenges and experiences of cervical cancer managers in Kenya and the group was generally homogeneous.

## Conclusion

Even though the results of this study may have limited generalized, they provide an insight into challenges faced in cervical cancer management from the health care providers’ perspective. Of interest were the negative attitudes towards screening procedure and care of patients with cervical cancer. This peculiar attitude of health care providers need urgent attention. With a large population with access to mobile phones and the positive attitude towards use of mobile phone in management of cervical cancer, the stakeholders need to exploit this opportunity. The negative attitude towards cervical cancer screening procedure may necessitate the stakeholders’ redress of the negative attitudes or provide HPV vaccination, which though expensive may be more culturally acceptable. There is need to address the low level of Knowledge, access to Internet and computers use by cervical cancer managers. Further research to establish how these factors interact and affect management of cervical cancer in Kenya is recommended.

## Competing interests

The authors declare there are no competing interests.

## Authors’ contribution

L WK designed the study, analyzed the data and drafted the manuscript. GM, AR, GP P took part in designing the study; and critically reviewed and revised the manuscript for important intellectual content. All authors read and approved the final manuscript.

## Supplementary Material

Additional file 1 **Appendix 1.** Interview Guide for Cervical Cancer Managers. Click here for file
