# Supplementary material for: An exploration of opportunities and challenges facing cervical cancer managers in Kenya
Source: BMC Res Notes. 2013 Apr 8;6:136. doi: 10.1186/1756-0500-6-136 (PMC3626574; doi:10.1186/1756-0500-6-136)
Supplement: Additional file 1 — Appendix 1. Interview Guide for Cervical Cancer Managers. [file 1756-0500-6-136-S1.doc]

**Appendix 1. Interview Guide for Cervical Cancer Managers**

**Instructions for research assistants/ Interviewer.**

***After introduction and signing of consent, Please position the tape recorder in a suitable place. Ensure that the room is as quite as possible and the voices are at a clear level of volume. The interview should not have interruptions however should there be interruption of any nature, pause the recorder until the interruption is sorted out and then continue with your interview.***

**Inform the respondent that,**

***The interview will take approximately 30 minutes to 1 hour.***

***Please answer the following questions as honestly possible. you are allowed to voice as many concerns/issues as you have during this interview.***

1. What is your Age in completed years.__________?
2. How long have you been involved in care of cervical cancer management_______
3. In what capacity have you been involved in care patients with cervical cancer? -------------
4. Have you been involved in caring for patients with other types of cancer? If so which ones? -----------
5. What challenges have you faced in management of cervical cancer patients?
6. How have you handled or dealt with the challenges?
7. What future challenges do you anticipate in management of cervical cancer clients?
8. Have you used computer technology in management of cervical cancer? If so what ways? If no, give reasons or challenges faced in use of computer in management of cancer.
9. Do you serve the internet in search of information or in management of cervical cancer? If so what sites have you visited in the last three months?
10. In what ways have you used the information obtained from the web?
11. In what ways would the internet be useful in management of cervical cancer in Kenya?
12. In what ways would the mobile phones be useful in management of cervical cancer in Kenya?
13. Apart from use of computers and internet in management of cervical cancer, what other measures would the key players put in place to ensure successful management of cervical cancer in Kenya.
14. Is there is any other comment or issue you would like to add regarding management of cervical cancer in Kenya today and in the future?

Thank you for your taking your time to take part in this study.

**Instructions for research assistants.**

**Ensure you play back the interview to the respondents and allow them to change or add any statement they may deem necessary.**
